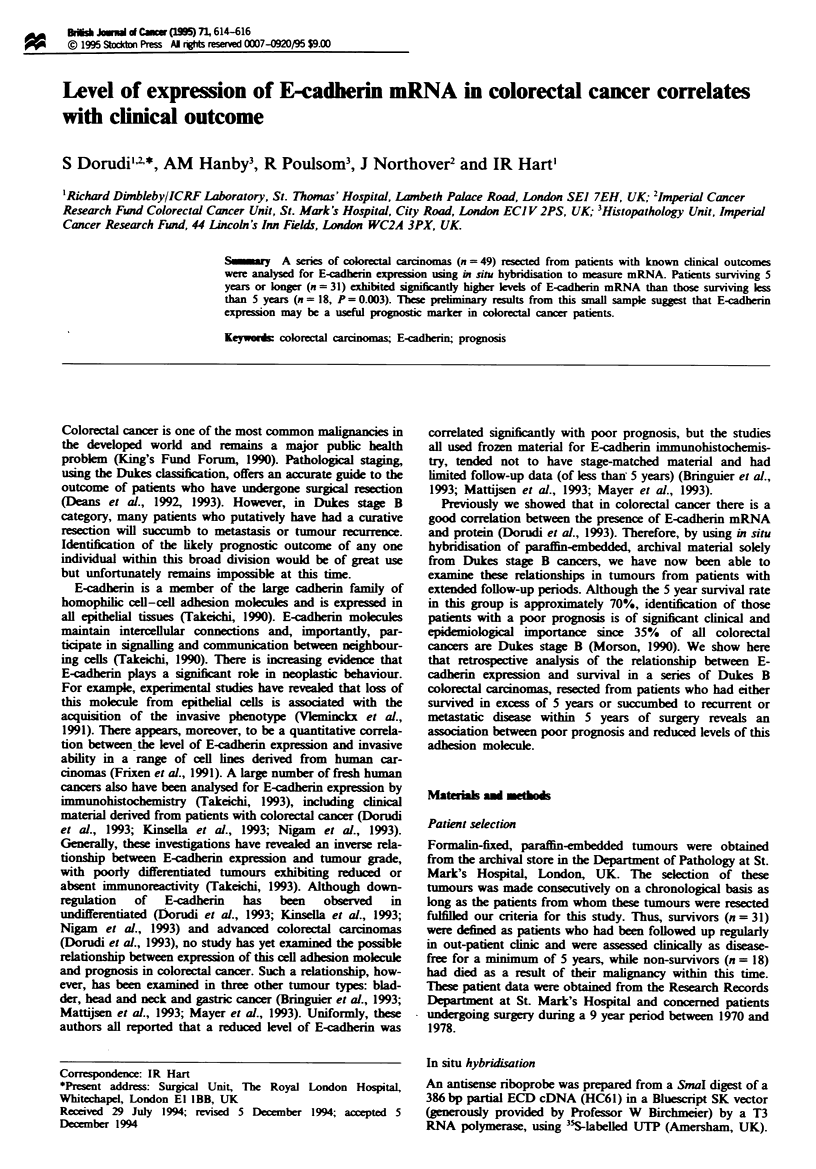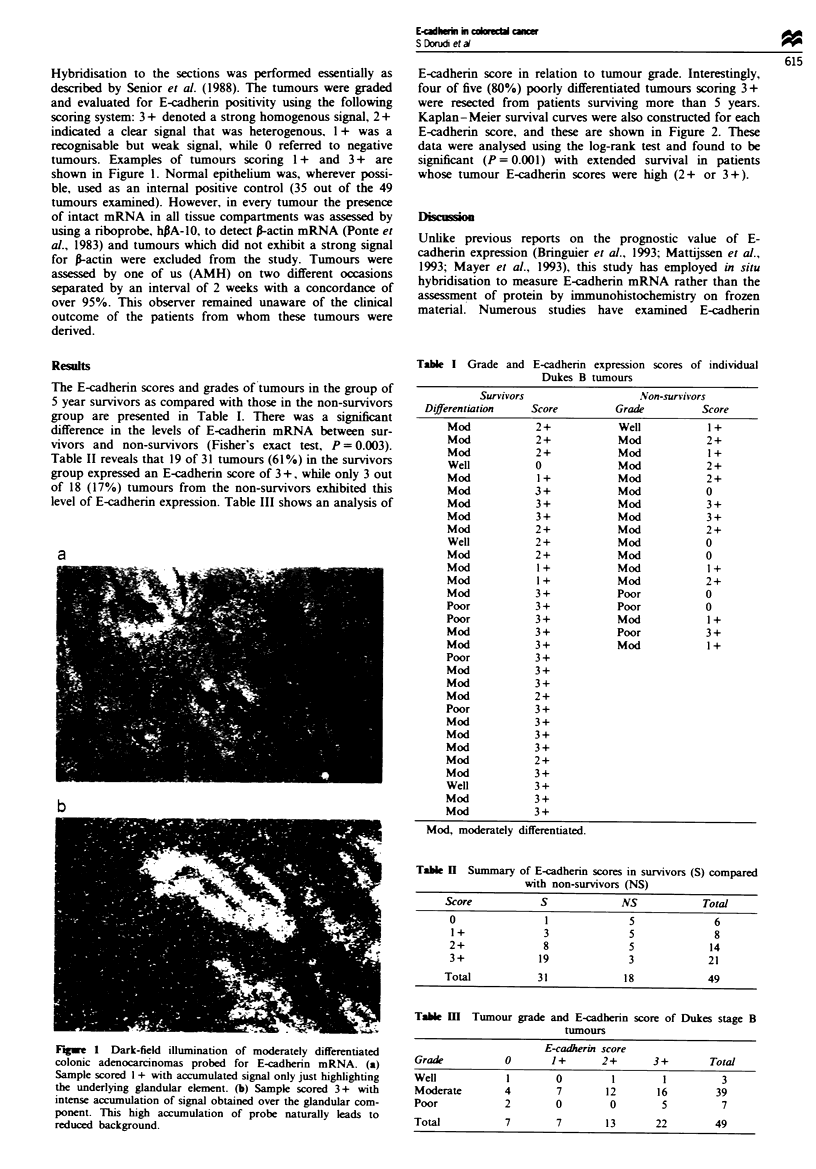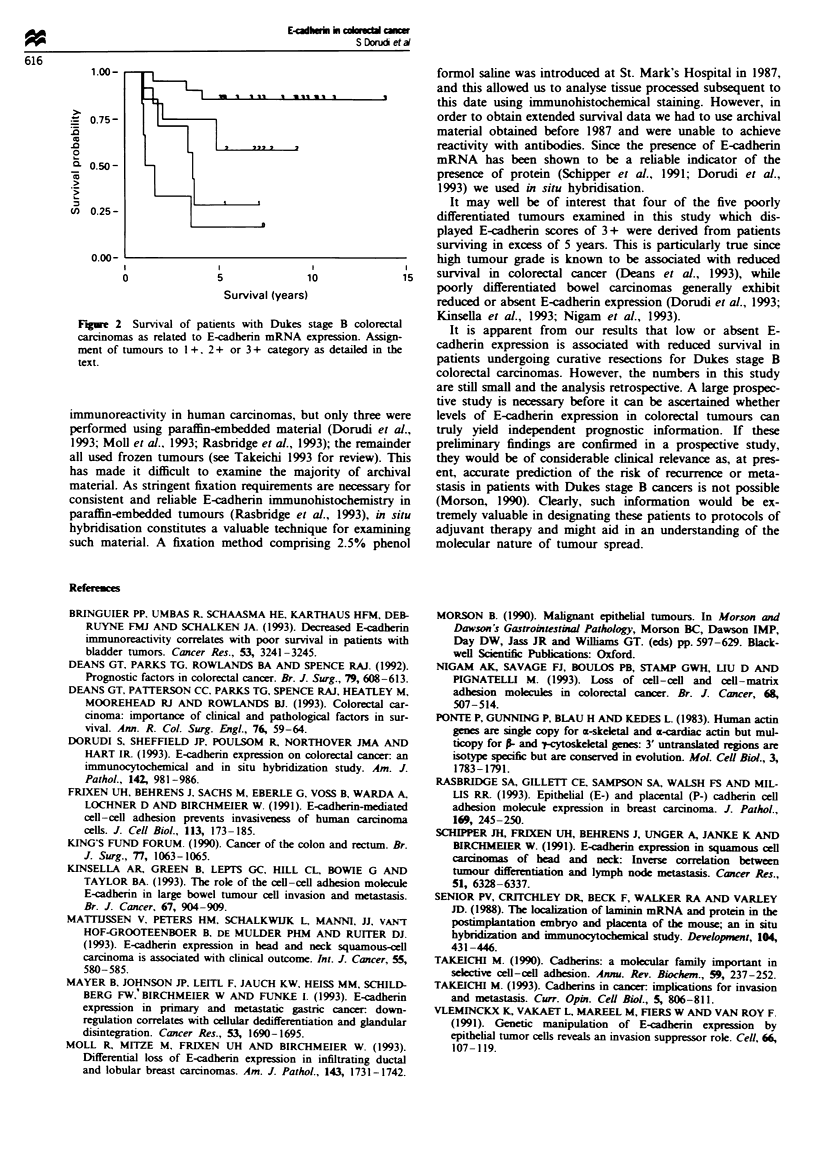# Level of expression of E-cadherin mRNA in colorectal cancer correlates with clinical outcome.

**DOI:** 10.1038/bjc.1995.119

**Published:** 1995-03

**Authors:** S. Dorudi, A. M. Hanby, R. Poulsom, J. Northover, I. R. Hart

**Affiliations:** Richard Dimbleby/ICRF Laboratory, St. Thomas' Hospital, London, UK.

## Abstract

**Images:**


© 1995 Stockton Press All rights reserved 0007-0920/95 $9.00

### Level of expression of E-cadherin mRNA in colorectal cancer correlates with clinical outcome

S Dorudi[1,2,*], AM Hanby[3], R Poulsom[3], J Northover[2] and IR Hart[1]

[1]*Richard Dimbleby/ICRF Laboratory, St. Thomas' Hospital, Lambeth Palace Road, London SE1 7EH, UK;* [2]*Imperial Cancer Research Fund Colorectal Cancer Unit, St. Mark's Hospital, City Road, London EC1V 2PS, UK;* [3]*Histopathology Unit, Imperial Cancer Research Fund, 44 Lincoln's Inn Fields, London WC2A 3PX, UK.*

**Summary** A series of colorectal carcinomas ($n = 49$) resected from patients with known clinical outcomes were analysed for E-cadherin expression using *in situ* hybridisation to measure mRNA. Patients surviving 5 years or longer ($n = 31$) exhibited significantly higher levels of E-cadherin mRNA than those surviving less than 5 years ($n = 18$, $P = 0.003$). These preliminary results from this small sample suggest that E-cadherin expression may be a useful prognostic marker in colorectal cancer patients.

**Keywords:** colorectal carcinomas; E-cadherin; prognosis

Colorectal cancer is one of the most common malignancies in the developed world and remains a major public health problem (King's Fund Forum, 1990). Pathological staging, using the Dukes classification, offers an accurate guide to the outcome of patients who have undergone surgical resection (Deans *et al.*, 1992, 1993). However, in Dukes stage B category, many patients who putatively have had a curative resection will succumb to metastasis or tumour recurrence. Identification of the likely prognostic outcome of any one individual within this broad division would be of great use but unfortunately remains impossible at this time.

E-cadherin is a member of the large cadherin family of homophilic cell-cell adhesion molecules and is expressed in all epithelial tissues (Takeichi, 1990). E-cadherin molecules maintain intercellular connections and, importantly, participate in signalling and communication between neighbouring cells (Takeichi, 1990). There is increasing evidence that E-cadherin plays a significant role in neoplastic behaviour. For example, experimental studies have revealed that loss of this molecule from epithelial cells is associated with the acquisition of the invasive phenotype (Vleminckx *et al.*, 1991). There appears, moreover, to be a quantitative correlation between the level of E-cadherin expression and invasive ability in a range of cell lines derived from human carcinomas (Frixen *et al.*, 1991). A large number of fresh human cancers also have been analysed for E-cadherin expression by immunohistochemistry (Takeichi, 1993), including clinical material derived from patients with colorectal cancer (Dorudi *et al.*, 1993; Kinsella *et al.*, 1993; Nigam *et al.*, 1993). Generally, these investigations have revealed an inverse relationship between E-cadherin expression and tumour grade, with poorly differentiated tumours exhibiting reduced or absent immunoreactivity (Takeichi, 1993). Although down-regulation of E-cadherin has been observed in undifferentiated (Dorudi *et al.*, 1993; Kinsella *et al.*, 1993; Nigam *et al.*, 1993) and advanced colorectal carcinomas (Dorudi *et al.*, 1993), no study has yet examined the possible relationship between expression of this cell adhesion molecule and prognosis in colorectal cancer. Such a relationship, however, has been examined in three other tumour types: bladder, head and neck and gastric cancer (Bringuier *et al.*, 1993; Mattijsen *et al.*, 1993; Mayer *et al.*, 1993). Uniformly, these authors all reported that a reduced level of E-cadherin was correlated significantly with poor prognosis, but the studies all used frozen material for E-cadherin immunohistochemistry, tended not to have stage-matched material and had limited follow-up data (of less than 5 years) (Bringuier *et al.*, 1993; Mattijsen *et al.*, 1993; Mayer *et al.*, 1993).

Previously we showed that in colorectal cancer there is a good correlation between the presence of E-cadherin mRNA and protein (Dorudi *et al.*, 1993). Therefore, by using *in situ* hybridisation of paraffin-embedded, archival material solely from Dukes stage B cancers, we have now been able to examine these relationships in tumours from patients with extended follow-up periods. Although the 5 year survival rate in this group is approximately 70%, identification of those patients with a poor prognosis is of significant clinical and epidemiological importance since 35% of all colorectal cancers are Dukes stage B (Morson, 1990). We show here that retrospective analysis of the relationship between E-cadherin expression and survival in a series of Dukes B colorectal carcinomas, resected from patients who had either survived in excess of 5 years or succumbed to recurrent or metastatic disease within 5 years of surgery reveals an association between poor prognosis and reduced levels of this adhesion molecule.

### Materials and methods

### Patient selection

Formalin-fixed, paraffin-embedded tumours were obtained from the archival store in the Department of Pathology at St. Mark's Hospital, London, UK. The selection of these tumours was made consecutively on a chronological basis as long as the patients from whom these tumours were resected fulfilled our criteria for this study. Thus, survivors ($n = 31$) were defined as patients who had been followed up regularly in out-patient clinic and were assessed clinically as disease-free for a minimum of 5 years, while non-survivors ($n = 18$) had died as a result of their malignancy within this time. These patient data were obtained from the Research Records Department at St. Mark's Hospital and concerned patients undergoing surgery during a 9 year period between 1970 and 1978.

### In situ *hybridisation*

An antisense riboprobe was prepared from a *SmaI* digest of a 386 bp partial ECD cDNA (HC61) in a Bluescript SK vector (generously provided by Professor W Birchmeier) by a T3 RNA polymerase, using $^{35}$S-labelled UTP (Amersham, UK).

Correspondence: IR Hart
*Present address: Surgical Unit, The Royal London Hospital, Whitechapel, London E1 1BB, UK
Received 29 July 1994; revised 5 December 1994; accepted 5 December 1994

Hybridisation to the sections was performed essentially as described by Senior *et al.* (1988). The tumours were graded and evaluated for E-cadherin positivity using the following scoring system: 3+ denoted a strong homogenous signal, 2+ indicated a clear signal that was heterogenous, 1+ was a recognisable but weak signal, while 0 referred to negative tumours. Examples of tumours scoring 1+ and 3+ are shown in Figure 1. Normal epithelium was, wherever possible, used as an internal positive control (35 out of the 49 tumours examined). However, in every tumour the presence of intact mRNA in all tissue compartments was assessed by using a riboprobe, hβA-10, to detect β-actin mRNA (Ponte *et al.*, 1983) and tumours which did not exhibit a strong signal for β-actin were excluded from the study. Tumours were assessed by one of us (AMH) on two different occasions separated by an interval of 2 weeks with a concordance of over 95%. This observer remained unaware of the clinical outcome of the patients from whom these tumours were derived.

### Results

The E-cadherin scores and grades of tumours in the group of 5 year survivors as compared with those in the non-survivors group are presented in Table I. There was a significant difference in the levels of E-cadherin mRNA between survivors and non-survivors (Fisher's exact test, $P = 0.003$). Table II reveals that 19 of 31 tumours (61%) in the survivors group expressed an E-cadherin score of 3+, while only 3 out of 18 (17%) tumours from the non-survivors exhibited this level of E-cadherin expression. Table III shows an analysis of E-cadherin score in relation to tumour grade. Interestingly, four of five (80%) poorly differentiated tumours scoring 3+ were resected from patients surviving more than 5 years. Kaplan–Meier survival curves were also constructed for each E-cadherin score, and these are shown in Figure 2. These data were analysed using the log-rank test and found to be significant ($P = 0.001$) with extended survival in patients whose tumour E-cadherin scores were high (2+ or 3+).

a b

**Figure 1** Dark-field illumination of moderately differentiated colonic adenocarcinomas probed for E-cadherin mRNA. (**a**) Sample scored 1+ with accumulated signal only just highlighting the underlying glandular element. (**b**) Sample scored 3+ with intense accumulation of signal obtained over the glandular component. This high accumulation of probe naturally leads to reduced background.

### Discussion

Unlike previous reports on the prognostic value of E-cadherin expression (Bringuier *et al.*, 1993; Mattijssen *et al.*, 1993; Mayer *et al.*, 1993), this study has employed *in situ* hybridisation to measure E-cadherin mRNA rather than the assessment of protein by immunohistochemistry on frozen material. Numerous studies have examined E-cadherin

**Table I** Grade and E-cadherin expression scores of individual Dukes B tumours

| *Survivors* | | *Non-survivors* | |
|---|---|---|---|
| *Differentiation* | *Score* | *Grade* | *Score* |
| Mod | 2+ | Well | 1+ |
| Mod | 2+ | Mod | 2+ |
| Mod | 2+ | Mod | 1+ |
| Well | 0 | Mod | 2+ |
| Mod | 1+ | Mod | 2+ |
| Mod | 3+ | Mod | 0 |
| Mod | 3+ | Mod | 3+ |
| Mod | 3+ | Mod | 3+ |
| Mod | 2+ | Mod | 2+ |
| Well | 2+ | Mod | 0 |
| Mod | 2+ | Mod | 0 |
| Mod | 1+ | Mod | 1+ |
| Mod | 1+ | Mod | 2+ |
| Mod | 3+ | Poor | 0 |
| Poor | 3+ | Poor | 0 |
| Poor | 3+ | Mod | 1+ |
| Mod | 3+ | Poor | 3+ |
| Mod | 3+ | Mod | 1+ |
| Poor | 3+ | | |
| Mod | 3+ | | |
| Mod | 3+ | | |
| Mod | 2+ | | |
| Poor | 3+ | | |
| Mod | 3+ | | |
| Mod | 3+ | | |
| Mod | 3+ | | |
| Mod | 2+ | | |
| Mod | 3+ | | |
| Well | 3+ | | |
| Mod | 3+ | | |
| Mod | 3+ | | |

Mod, moderately differentiated.

**Table II** Summary of E-cadherin scores in survivors (S) compared with non-survivors (NS)

| *Score* | *S* | *NS* | *Total* |
|---|---|---|---|
| 0 | 1 | 5 | 6 |
| 1+ | 3 | 5 | 8 |
| 2+ | 8 | 5 | 14 |
| 3+ | 19 | 3 | 21 |
| Total | 31 | 18 | 49 |

**Table III** Tumour grade and E-cadherin score of Dukes stage B tumours

| | *E-cadherin score* | | | | |
|---|---|---|---|---|---|
| *Grade* | *0* | *1+* | *2+* | *3+* | *Total* |
| Well | 1 | 0 | 1 | 1 | 3 |
| Moderate | 4 | 7 | 12 | 16 | 39 |
| Poor | 2 | 0 | 0 | 5 | 7 |
| Total | 7 | 7 | 13 | 22 | 49 |

**Figure 2** Survival of patients with Dukes stage B colorectal carcinomas as related to E-cadherin mRNA expression. Assignment of tumours to 1+, 2+ or 3+ category as detailed in the text.

immunoreactivity in human carcinomas, but only three were performed using paraffin-embedded material (Dorudi *et al.*, 1993; Moll *et al.*, 1993; Rasbridge *et al.*, 1993); the remainder all used frozen tumours (see Takeichi 1993 for review). This has made it difficult to examine the majority of archival material. As stringent fixation requirements are necessary for consistent and reliable E-cadherin immunohistochemistry in paraffin-embedded tumours (Rasbridge *et al.*, 1993), *in situ* hybridisation constitutes a valuable technique for examining such material. A fixation method comprising 2.5% phenol formol saline was introduced at St. Mark's Hospital in 1987, and this allowed us to analyse tissue processed subsequent to this date using immunohistochemical staining. However, in order to obtain extended survival data we had to use archival material obtained before 1987 and were unable to achieve reactivity with antibodies. Since the presence of E-cadherin mRNA has been shown to be a reliable indicator of the presence of protein (Schipper *et al.*, 1991; Dorudi *et al.*, 1993) we used *in situ* hybridisation.

It may well be of interest that four of the five poorly differentiated tumours examined in this study which displayed E-cadherin scores of 3+ were derived from patients surviving in excess of 5 years. This is particularly true since high tumour grade is known to be associated with reduced survival in colorectal cancer (Deans *et al.*, 1993), while poorly differentiated bowel carcinomas generally exhibit reduced or absent E-cadherin expression (Dorudi *et al.*, 1993; Kinsella *et al.*, 1993; Nigam *et al.*, 1993).

It is apparent from our results that low or absent E-cadherin expression is associated with reduced survival in patients undergoing curative resections for Dukes stage B colorectal carcinomas. However, the numbers in this study are still small and the analysis retrospective. A large prospective study is necessary before it can be ascertained whether levels of E-cadherin expression in colorectal tumours can truly yield independent prognostic information. If these preliminary findings are confirmed in a prospective study, they would be of considerable clinical relevance as, at present, accurate prediction of the risk of recurrence or metastasis in patients with Dukes stage B cancers is not possible (Morson, 1990). Clearly, such information would be extremely valuable in designating these patients to protocols of adjuvant therapy and might aid in an understanding of the molecular nature of tumour spread.

### References

BRINGUIER PP, UMBAS R, SCHAASMA HE, KARTHAUS HFM, DEBRUYNE FMJ AND SCHALKEN JA. (1993). Decreased E-cadherin immunoreactivity correlates with poor survival in patients with bladder tumors. *Cancer Res.*, **53**, 3241-3245.

DEANS GT, PARKS TG, ROWLANDS BA AND SPENCE RAJ. (1992). Prognostic factors in colorectal cancer. *Br. J. Surg.*, **79**, 608-613.

DEANS GT, PATTERSON CC, PARKS TG, SPENCE RAJ, HEATLEY M, MOOREHEAD RJ AND ROWLANDS BJ. (1993). Colorectal carcinoma: importance of clinical and pathological factors in survival. *Ann. R. Col. Surg. Engl.*, **76**, 59-64.

DORUDI S, SHEFFIELD JP, POULSOM R, NORTHOVER JMA AND HART IR. (1993). E-cadherin expression on colorectal cancer: an immunocytochemical and in situ hybridization study. *Am. J. Pathol.*, **142**, 981-986.

FRIXEN UH, BEHRENS J, SACHS M, EBERLE G, VOSS B, WARDA A, LOCHNER D AND BIRCHMEIER W. (1991). E-cadherin-mediated cell-cell adhesion prevents invasiveness of human carcinoma cells. *J. Cell Biol.*, **113**, 173-185.

KING'S FUND FORUM. (1990). Cancer of the colon and rectum. *Br. J. Surg.*, **77**, 1063-1065.

KINSELLA AR, GREEN B, LEPTS GC, HILL CL, BOWIE G AND TAYLOR BA. (1993). The role of the cell-cell adhesion molecule E-cadherin in large bowel tumour cell invasion and metastasis. *Br. J. Cancer*, **67**, 904-909.

MATTIJSSEN V, PETERS HM, SCHALKWIJK L, MANNI, JJ, VANT HOF-GROOTEENBOER B, DE MULDER PHM AND RUITER DJ. (1993). E-cadherin expression in head and neck squamous-cell carcinoma is associated with clinical outcome. *Int. J. Cancer*, **55**, 580-585.

MAYER B, JOHNSON JP, LEITL F, JAUCH KW, HEISS MM, SCHILDBERG FW, BIRCHMEIER W AND FUNKE I. (1993). E-cadherin expression in primary and metastatic gastric cancer: down-regulation correlates with cellular dedifferentiation and glandular disintegration. *Cancer Res.*, **53**, 1690-1695.

MOLL R, MITZE M, FRIXEN UH AND BIRCHMEIER W. (1993). Differential loss of E-cadherin expression in infiltrating ductal and lobular breast carcinomas. *Am. J. Pathol.*, **143**, 1731-1742.

MORSON B. (1990). Malignant epithelial tumours. In *Morson and Dawson's Gastrointestinal Pathology*, Morson BC, Dawson IMP, Day DW, Jass JR and Williams GT. (eds) pp. 597-629. Blackwell Scientific Publications: Oxford.

NIGAM AK, SAVAGE FJ, BOULOS PB, STAMP GWH, LIU D AND PIGNATELLI M. (1993). Loss of cell-cell and cell-matrix adhesion molecules in colorectal cancer. *Br. J. Cancer*, **68**, 507-514.

PONTE P, GUNNING P, BLAU H AND KEDES L. (1983). Human actin genes are single copy for α-skeletal and α-cardiac actin but multicopy for β- and γ-cytoskeletal genes: 3' untranslated regions are isotype specific but are conserved in evolution. *Mol. Cell Biol.*, **3**, 1783-1791.

RASBRIDGE SA, GILLETT CE, SAMPSON SA, WALSH FS AND MILLIS RR. (1993). Epithelial (E-) and placental (P-) cadherin cell adhesion molecule expression in breast carcinoma. *J. Pathol.*, **169**, 245-250.

SCHIPPER JH, FRIXEN UH, BEHRENS J, UNGER A, JANKE K AND BIRCHMEIER W. (1991). E-cadherin expression in squamous cell carcinomas of head and neck: Inverse correlation between tumour differentiation and lymph node metastasis. *Cancer Res.*, **51**, 6328-6337.

SENIOR PV, CRITCHLEY DR, BECK F, WALKER RA AND VARLEY JD. (1988). The localization of laminin mRNA and protein in the postimplantation embryo and placenta of the mouse; an in situ hybridization and immunocytochemical study. *Development*, **104**, 431-446.

TAKEICHI M. (1990). Cadherins: a molecular family important in selective cell-cell adhesion. *Annu. Rev. Biochem.*, **59**, 237-252.

TAKEICHI M. (1993). Cadherins in cancer: implications for invasion and metastasis. *Curr. Opin. Cell Biol.*, **5**, 806-811.

VLEMINCKX K, VAKAET L, MAREEL M, FIERS W AND VAN ROY F. (1991). Genetic manipulation of E-cadherin expression by epithelial tumor cells reveals an invasion suppressor role. *Cell*, **66**, 107-119.